# Diagnostic and prognostic performance of urine ubiquitin carboxy-terminal hydrolase L1 across multiple acute brain injury types – A longitudinal prospective cohort study

**DOI:** 10.1016/j.bas.2024.104173

**Published:** 2024-12-24

**Authors:** Santtu Hellström, Antti Sajanti, Aditya Jhaveri, Abhinav Srinath, Carolyn Bennett, Ying Cao, Fredrika Koskimäki, Johannes Falter, Janek Frantzén, Seán B. Lyne, Tomi Rantamäki, Riikka Takala, Jussi P. Posti, Susanna Roine, Sulo Kolehmainen, Miro Jänkälä, Jukka Puolitaival, Romuald Girard, Melissa Rahi, Jaakko Rinne, Eero Castrén, Janne Koskimäki

**Affiliations:** a Neurocenter, Department of Neurosurgery, Turku University Hospital and University of Turku, P.O. Box 52, FI-20521, Turku, Finland; b Neurovascular Surgery Program, Section of Neurosurgery, The University of Chicago Medicine and Biological Sciences, Chicago, IL, 60637, USA; c Department of Radiation Oncology, Kansas University Medical Center, Kansas City, KS, 66160, USA; d Department of Neurosurgery, University Medical Center of Regensburg, Regensburg, 93042, Germany; e Neurocenter, Acute Stroke Unit, Turku University Hospital, P.O. Box 52, FI-20521, Turku, Finland; f Department of Neurosurgery, Brigham and Women's Hospital, Harvard Medical School, Boston, MA, USA; g Laboratory of Neurotherapeutics, Drug Research Program, Division of Pharmacology and Pharmacotherapy, Faculty of Pharmacy, University of Helsinki, P.O. Box 56, FI-00014, Helsinki, Finland; h SleepWell Research Program, Faculty of Medicine, University of Helsinki, P.O. Box 63, FI-00014, Helsinki, Finland; i Perioperative Services, Intensive Care and Pain Medicine and Department of Anaesthesiology and Intensive Care, Turku University Hospital and University of Turku, P.O. Box52, FI-20521, Turku, Finland; j Neuroscience Center, HiLIFE, University of Helsinki, P.O. Box 63, FI-00014, Helsinki, Finland; k Department of Neurosurgery, Oulu University Hospital, Box 25, OYS, 90029, Finland

**Keywords:** Brain injury, UCHL1, Urine, Outcome, Stroke, Traumatic brain injury

## Abstract

**Introduction:**

Ubiquitin carboxy-terminal hydrolase L1 (UCH-L1) is recognized as a diagnostic and prognostic blood biomarker for traumatic brain injury (TBI). This study aimed to evaluate whether UCH-L1 concentrations measured in patients' urine post-injury could serve as a diagnostic or prognostic biomarker for outcomes in various types of acute brain injuries (ABI).

**Material and methods:**

This pilot study included 46 ABI patients: aneurysmal subarachnoid hemorrhage (n = 22), ischemic stroke (n = 16), and traumatic brain injury (n = 8), along with three healthy controls. Urine samples were collected at early (1.50 ± 0.70 days) and late (9.17 ± 3.40 days) periods post-admission. UCH-L1 and creatinine levels were quantified using ELISA. UCH-L1 concentrations were compared to functional outcomes (modified Rankin Scale, mRS) and dichotomized into favorable (mRS 0–3) and unfavorable (mRS 4–6) groups. Non-parametric statistical tests and ROC analysis was performed.

**Results:**

UCH-L1 concentrations in healthy controls were significantly lower compared to both early and late samples after ABI (p ≤ 0.001). The diagnostic performance of urine UCH-L1 at early timepoint showed excellent discriminatory ability, with AUC of 97.6% (95% CI: 93.0–100, p = 0.006 (sensitivity 98%, specificity 100%). Urine UCH-L1 concentrations, both with and without creatinine normalization, did not distinguish between favorable and unfavorable outcomes in either early (p = 0.88 and p = 0.36) or late samples (p = 0.98 and p = 0.30) in any types of ABI.

**Discussion and conclusions:**

Although UCH-L1 concentrations in urine did not differentiate between favorable and unfavorable outcomes, a significant difference was observed between healthy subjects and ABI patients. This finding underscores the significant diagnostic utility of urine UCH-L1 concentrations, regardless of the type of acute brain injury.

## Highlights

• Urine UCH-L1 levels were higher in brain injury patients than controls supporting diagnostic potential across injury types.
• UCH-L1 levels in urine did not distinguish between favorable and unfavorable outcomes, indicating limited prognostic value.
• The findings encourage further research to validate urine UCH-L1 as an accessible diagnostic tool for acute brain injuries.

## 1 Introduction

Acute brain injuries (ABI), such as traumatic brain injury (TBI), ischemic stroke (IS), and aneurysmal subarachnoid hemorrhage (aSAH), are major causes of mortality and disability worldwide (Feigin et al., 2022; Capizzi et al., 2020). These injuries can lead to significant alterations in brain anatomy and function, resulting in temporary or permanent neurological deficits irrespective of the underlying cause (Galgano et al., 2017; Long et al., 2017; Chen et al., 2012). Although treatment strategies for these conditions have advanced significantly, predicting clinical outcomes remains challenging due to the wide variability in outcomes (Rabinstein, 2020; Hannah et al., 2021; van Essen et al., 2022; Bendszus et al., 2023; Molyneux et al., 2005).

After an initial injury to the brain, a series of complex biochemical and cellular processes are activated within the central nervous system (CNS), which ultimately leads to dysfunction and apoptosis of neurons and supporting cells. These dysregulated processes and pathways are potential targets for rehabilitation interventions (Pischiutta et al., 2018). The concept of neuroplasticity is of utmost importance in neuronal rehabilitation, as it promotes structural and functional reorganization of the nervous system in response to stimuli (Coleman et al., 2017; Stewart and Cramer, 2017; Belagaje, 2017). Recently, there has been a growing interest in understanding the activity of biomolecules and their associated receptors, which may play a role in neuronal plasticity and rehabilitation. Biomarkers related to the pathophysiology of brain injuries offer new possibilities for addressing diagnostic, prognostic, and therapeutic questions concerning such injuries (Mondello et al., 2012a; Liu et al., 2010).

One significant biomarker in the central nervous system is ubiquitin carboxy-terminal hydrolase L1 (UCH-L1), also known as neuronal-specific protein gene product 9.5 (PGP9.5) (Jackson and Thompson, 1981). UCH-L1 is one of the most abundant soluble proteins in the brain (Jackson and Thompson, 1981), representing 1–2 % of the soluble proteins in the brain (Wilson et al., 1988). Moreover, UCH-L1 belongs to the ubiquitin carboxy-terminal hydrolase family of deubiquitinating enzymes. Depending on the context, UCH-L1 functions by both detaching and adding ubiquitin to proteins, thus playing a significant role in the clearance of excessive or defective proteins (oxidized or misfolded) (Liu et al., 2010; Gong and Leznik, 2007). Reduced UCH-L1 activity has been linked to neurodegenerative diseases such as Alzheimer's and Parkinson's disease (Choi et al., 2004). An increase in the concentration of UCH-L1 has been observed in epilepsy, with levels rising following the occurrence of seizures (Mondello et al., 2012b). UCH-L1 also exhibits expression outside the central nervous system, such as in ovary, testis and kidneys (Brophy et al., 2011; Wilkinson et al., 1989).

There is significant interest in the potential of UCH-L1 for diagnostic assessment of brain injuries. In numerous animal and human studies, the biomarker has been found to increase after brain injury in both cerebrospinal fluid and blood. In the bloodstream, UCH-L1 is measurable within 1 h of injury, peaks 8 h after injury, and has a half-life of 7–9 h after injury (Brophy et al., 2011; Papa et al., 2016). Therefore, UCH-L1 has been recognized for its diagnostic value in acute situations (Liu et al., 2010; Papa et al., 2010; Kobeissy et al., 2006; Ren et al., 2013, 2016). UCH-L1 has been certified by the U.S. Food and Drug Administration (FDA) as a biomarker, currently only in the blood, which clinicians may use to assess the need for head CT scans in cases of mild TBI 12–24 h post-injury (Papa et al., 2012; Commissioner O of the. et al., 2020). However, biomarker samples taken from urine have been studied significantly less. Following brain injury, the permeability of the blood-brain barrier (BBB) increases. UCH-L1, identified as a small molecule (27 kDa), is known to pass easily through the BBB and is freely filtered in the kidneys into urine. Consequently, UCH-L1 can be measured from both blood and urine (Helbok et al., 2018; Blyth et al., 2011). The advantage of a urinary biomarker would be the excellent accessibility and non-invasiveness of sample collection, which could be utilized, for example, in sports-related injuries or in nursing homes that are not equipped for blood sampling. In a recent study, it was observed that at one point in time (<96 h after insult), UCH-L1 appeared in absolutely higher concentrations in the urine samples of patients who had suffered acute IS or intracerebral hemorrhage compared to a healthy control group (Kohlhase et al., 2023). Although UCH-L1, when normalized to creatinine, was not a significant indicator of brain injuries in that study, this finding sparks interest in further research (Kohlhase et al., 2023).

In this longitudinal prospective cohort study, we examine the levels of UCH-L1 in urine samples collected at different timepoints and investigate its association with outcomes based on the modified Rankin Scale (mRS) measured three months later. By emphasizing the temporal and functional components, we aim to determine whether changes in UCH-L1 concentrations over time can provide insight into the progression and potential outcomes of various acute brain injuries. Diagnostic performance of UCH-L1 was also evaluated comparing studied ABIs to healthy subjects.

## 2 Materials and methods

### 2.1 Study methods and participants

**Fig. 1:**
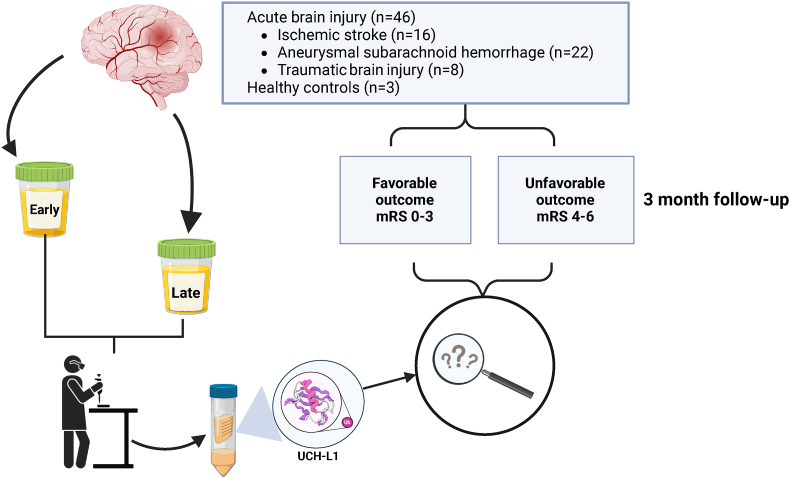
**Flow chart of the investigation.** The longitudinal prospective cohort (n = 46) consisted of consecutively enrolled patients with ischemic stroke (IS) (n = 16), aneurysmal subarachnoid hemorrhage (aSAH) (n = 22), and traumatic brain injury (TBI) (n = 8). Patients were categorized based on their modified Rankin Scale (mRS) scores into favorable (mRS = 0–3) and unfavorable (mRS = 4–6) outcome groups. Urine samples were collected at two timepoints, early (1.50 ± 0.70 days) and late (9.17 ± 3.40 days) post-admission. Three healthy controls were included in the study. The concentration of ubiquitin carboxy-terminal hydrolase L1 (UCH-L1) was measured from urine samples and adjusted for urine creatinine concentration. Statistical analyses were performed to study the prognostic performance of the measured urine biomarker UCH-L1.

We carried out a longitudinal, observational, prospective cohort study at the University Hospital of Turku, Finland, collecting urine samples from patients who suffered an ABI and were treated there between 2017 and 2019 (Fig. 1). The study included patients with aSAH, IS (thrombotic, embolic, or cryptogenic), or TBI leading to an acute subdural hematoma with or without concomitant intraparenchymal injuries that required surgical intervention. All participants were adults over 18 years of age who provided informed consent. ABI patients received treatment based on institutional protocols that adhere to the latest guidelines for specific brain injuries (Connolly et al., 2012; Powers et al., 2019; Carney et al., 2017). Urine samples were collected early (1.50 ± 0.70 days) and late (9.17 ± 3.40 days) post-injury. A total of 74 patients were recruited for the study, with 46 patients having both early and late urine samples available, forming the study cohort. Eleven patients declined participation. One patient gave consent but decided later to withdraw from the study, thus samples and other data were not used in the study. The study cohort of 46 patients was divided into three groups: aSAH (n = 22), IS (n = 16), and TBI (n = 8), with an additional three healthy controls (n = 3) without neurological diseases. Patients were classified into two outcome categories using the mRS: favorable (mRS 0–3) and unfavorable (mRS 4–6). Outcomes were assessed after a three-month follow-up period; aSAH patients attended an outpatient clinic, while IS and TBI patients participated in structured telephone interviews. No patients were lost to follow-up during the three months.

### 2.2 External healthy cohort

From the previously published article by Kohlhase et al. (2023), we used urine UCH-L1 concentration data from healthy control subjects (n = 10) to compare with UCH-L1 concentrations in our study. Lastly, we imputed this data into the receiver operating characteristics (ROC) analysis to validate our diagnostic model (2.4. Statistical analyses).

### 2.3 Assessment of biomarkers

Urine samples were collected and stored according to the Urine and Kidney Proteome Project Standards (Yamamoto, 2010). Samples were pulled in a sterile syringe from the catheter, centrifuged at 1000 g for 10 min to remove cells and debris, and stored in 10 mL aliquots at −80 °C. Samples were given de-identified codes. UCH-L1 concentrations of the urine samples were measured using commercially available enzyme-linked immunosorbent assays (Invitrogen®, Catalog number: EH475RBX10, analytical sensitivity 0.82 ng/ml, assay range 0.82–200 ng/ml). Urinary creatinine measurements were performed using the Creatinine Urinary Detection Kit (Invitrogen® Catalog number: EIACUN, analytical sensitivity 0.019 mg/dl, assay range 0.3–20 mg/dl).

Samples were diluted 1:4 and loaded in parallel duplicate wells, with measurements obtained following the manufacturer's instructions. Absorbance values were read (450 nm for UCH-Ll and 490 nm for creatinine) using a Varioskan® Flash analyzer with SkanIt Software version 2.4.3 RE. For all ELISA tests, samples and standards were measured in duplicate, and the means of the duplicates were used for statistical analyses. One plate per disease group was used for each biomarker, resulting in a total of six ELISAs (three UCH-L1 plates and three creatinine plates). An experienced researcher, blinded to the subjects' clinical outcomes, performed the plate loading. Sample concentrations were estimated using a four-parameter logistic regression analysis, following the ELISA kit guidelines. There was no evidence of a batch effect according to the analysis of the standardized concentration controls, as the control concentrations (three different concentrations per plate) were consistent across all plates (coefficients variability (CV) < 10%).

The approach in the previous literature has been to investigate the association between UCH-L1 and creatinine (Crea) concentrations (ng/mg) because urinary protein biomarkers as a protein-to-creatinine ratio may help to mitigate variability and improve the accuracy, consistency, and clinical relevance of the findings (Kohlhase et al., 2023; Shepheard et al., 2014). In this study, when referring to UCH-L1/Crea, we denote the ratio of UCH-L1 (ng) to Crea (mg). We also studied UCH-L1 concentrations without normalization (UCH-L1 ng/dl) as has been the case in previous study.

### 2.4 Statistical analyses

Urine UCH-L1 data did not meet the criteria for normal distribution; thus, non-parametric tests were used. To compare the differences in UCH-L1 between patients with favorable outcomes (mRS scores of 0–3) and those with unfavorable outcomes (mRS scores of 4–6), we employed a Mann-Whitney *U* test. Temporal changes in the UCH-L1 were assessed using the paired Wilcoxon matched-pairs rank test. To study concentration differences of UCH-L1 between the three disease groups the Kruskal-Wallis test was used. Age and sex comparisons between healthy and favorable, and healthy and unfavorable outcome groups were performed using the unpaired *t*-test for age and Fisher's exact test for sex. A chi-square test was conducted to compare the proportions of different types of brain injuries between the favorable and unfavorable outcome groups. Dunnet's T3 multiple comparison test was performed to study urine UCH-L1 differences between healthy control patients, external healthy control patients previously published (Kohlhase et al., 2023), and early ABI patients. ROC analysis was performed to test the diagnostic performance of UCH-L1 and the area under the curve (AUC) was calculated with and without an external validation cohort. The Youden method was applied to determine the optimum sensitivity and specificity (Youden, 1950). We identified potential outliers in both cohorts and excluded them from the analysis. This was achieved using the widely used ROUT method, with a false discovery rate set at Q = 1% (Motulsky and Brown, 2006). All data analyses were conducted using SAS 9.4 (SAS Institute Inc., 2016; Cary, NC, US) and Prism 9.4.1 (GraphPad Software, LLC). P < 0.05 was interpreted as a statistically significant result. In Dunnet's T3 multiple comparison test, p-values were adjusted for comparisons and adjusted p < 0.05 was interpreted as a statistically significant result.

### 2.5 Study approval and ethics

The study was approved by the Turku University Hospital Institutional Review Board and Ethics Committee (T291/2016) and was conducted in accordance with the Declaration of Helsinki and its subsequent amendments. All participants provided written informed consent to participate in the study. For patients unable to provide consent due to severe acute illness, a legal trustee authorized participation on their behalf. The ethical principles guiding the Institutional Review Board are consistent with the Belmont Report, the Declaration of Helsinki, and comply with Finnish legislation and regulations.

## 3 Results

### 3.1 Demographics of studied patients

Forty-six urine samples were collected from three distinct groups of ABI patients at two timepoints (aSAH (n = 22, 47.8%), TBI (n = 8, 17.4%), and IS (n = 16, 34.8%)) (Fig. 1). Additionally, samples were obtained from three healthy controls. The age distribution for the entire patient cohort ranged from 23 to 75 years. The mean age for ABI patients was 57.8 ± 13.3 years, compared to 47.33 ± 19.09 years for the healthy controls. The studied outcome groups, and healthy controls were balanced in terms of age and gender (Table 1).

**Table 1:** Characteristics and concentrations of urine UCH-L1/Crea from the acute brain injury cohort (n = 46) and healthy individuals modified Rankin scale (mRS). Favorable mRS 0–3, Unfavorable mRS 4–6.

Variables	Favorable (n = 30)	Unfavorable (n = 16)	p-value
**Age in years**			0.7283
Mean ± SD	57.3 ± 13.3	58.8 ± 13.7	
Min–Max	23.0–75.0	34.0–74.0	
Median (IQR)	60.0 (48.0–68.0)	65.0 (45.0–70.0)	
**Sex**			0.9783
Male	17 (56.7)	9 (56.3)	
Female	13 (43.3)	7 (43.8)	
**Type of brain injury**			0.0373^a^
aSAH	13 (43.3)	9 (56.3)	
TBI	3 (10.0)	5 (31.3)	
IS	14 (46.7)	2 (12.5)	
**UCH-L1/Crea Early (ng/mg)**			0.8848
Mean ± SD	303.6 ± 316.0	244.9 ± 185.3	
Min–Max	2.77–1033	36.40–605.7	
Median (IQR)	138.8 (69.79–461.2)	190.1 (73.10–397.3)	
**UCH-L1/Crea Late (ng/mg)**			0.9675
Mean ± SD	421.4 ± 437.4	408.3 ± 462.5	
Min–Max	10.63–1424	24.70–1633	
Median (IQR)	309.9 (60.47–666.1)	248.7 (82.18–725.4)	

**UCH-L1/Crea Healthy (ng/mg)**	**Healthy (n = 3)**		>0.05^b^
Mean ± SD	313.0 ± 406.3		
Min–Max	55.18–781.3		
Median (IQR)	102.5 (55.18–781.3)		
**Age in years**			>0.05^c^
Mean ± SD	47.33 ± 19.09		
Min–Max	33.00–69.00		
Median (IQR)	40.00 (33.00–69.00)		
**Sex**			>0.05^c^
Male	1 (33.33)		
Female	2 (66.67)		

a Chi-square test.

b Statistical comparisons of UCH-L1/Crea between healthy versus favorable, and healthy versus unfavorable outcome groups (both comparisons p > 0.05). Mann-Whitney *U* test.

c Statistical comparisons of age and sex between healthy versus favorable, and healthy versus unfavorable outcome groups (both comparisons p > 0.05). Unpaired *t*-test (age) and Fisher's exact test (sex).

As for the outcomes, 65.2% (30/46) of the patients had favorable outcomes, while 34.8% (16/46) had unfavorable outcomes (Table 1). Among the patients with favorable outcomes, the distribution of brain injuries was 13 aSAH (43.3%), three TBI (10%), and 14 IS (46.7%). For patients with unfavorable outcomes, nine had aSAH (56.3%), five had TBI (31.3%), and two had IS (12.5%). Detailed disease-specific characteristics are provided in the supplement (**Supplemental material and** Supplemental Table S1).

### 3.2 UCH-L1 profiles and disease characteristics in urine among various ABIs

We studied specific disease characteristics, along with age and sex, to determine their correlations with UCH-L1 levels. Age and sex did not significantly correlate with UCH-L1 levels in ABI patients (p = 0.8169 and p = 0.6522, respectively) (Supplemental Fig. S1). In ischemic stroke, UCH-L1 levels showed a non-statistically significant trend of correlation with stroke type (p = 0.0666) but not with volume or location (p = 0.8852 and p = 0.4979) (Supplemental Fig. S2). UCH-L1 levels were inversely correlated with delayed cerebral ischemia in aSAH patients (p = 0.0202) (Supplemental Fig. S3). In TBI, UCH-L1 levels correlated with the volume of acute subdural hematoma (p = 0.0011) (Supplemental Fig. S4).

Furthermore, our results showed similar concentrations among the same outcome and timepoint groups in UCH-L1 profiles irrespective of the disease group aSAH, IS, and TBI patients, with no statistically significant differences in concentrations (Supplemental Fig. S5). This observation of similarity guided our analysis to identify common signatures in the studied ABIs.

### 3.3 UCH-L1 in urine does not associate with outcome

**Fig. 2:**
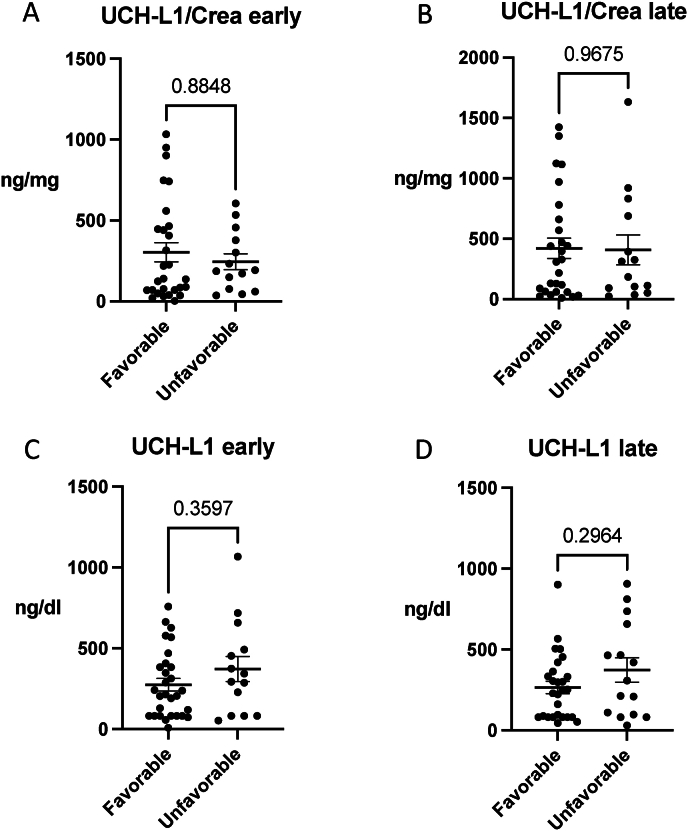
**Urine ubiquitin carboxy-terminal hydrolase L1 (UCH-L1) concentrations in acute brain injury (ABI) patients with (UCH-L1/Crea) and without (UCH-L1) normalization to creatinine testing prognostic associations with favorable and unfavorable outcome.** A) In the early timepoint, urine UCH-L1/Crea concentration was not able to differentiate outcome groups (p = 0.8848). B) In the late timepoint, urine UCH-L1/Crea concentration was not able to differentiate outcome groups (p = 0.9675). C) We identified an absolute increase in the urine UCH-L1 concentration in the early unfavorable group, but that increase was not statistically significant (p = 0.3597). D) A similar absolute increase in the urine UCH-L1 was observed in the late unfavorable group, but statistically significant change was not reached (p = 0.2964). Early timepoint = 1.50 ± 0.70 days; late timepoint 9.17 ± 3.40 days. Favorable = modified Ranking Scale (mRS) 0–3; unfavorable outcome = mRS 4–6. The data represent mean ± SEM (UCH-L1/Crea, ng/mg) or absolute UCH-L1 concentration mean ± SEM ng/dl. Mann-Whitney *U* test.

The mean UCH-L1/Crea concentration in early urine samples was 303.6 ± 316.0 ng/mg for the favorable outcome group and 244.9 ± 185.3 ng/mg for the unfavorable outcome group (p = 0.88) (Table 1, Fig. 2A and B). In late urine samples, the mean UCH-L1/Crea concentration was 421.4 ± 437.4 ng/mg for the favorable group and 408.3 ± 462.5 ng/mg for the unfavorable group (p = 0.97). We further analyzed the UCH-L1 levels without normalization. Similarly, we were not able to show statistically significant discriminatory power between favorable and unfavorable outcome groups (early p = 0.36 and late p = 0.30) but the unfavorable group in early and late timepoints had observable higher absolute levels of UCHL1 in urine than in the favorable groups (Fig. 2C and D).

### 3.4 Temporal changes of UCH-L1 in urine

**Fig. 3:**
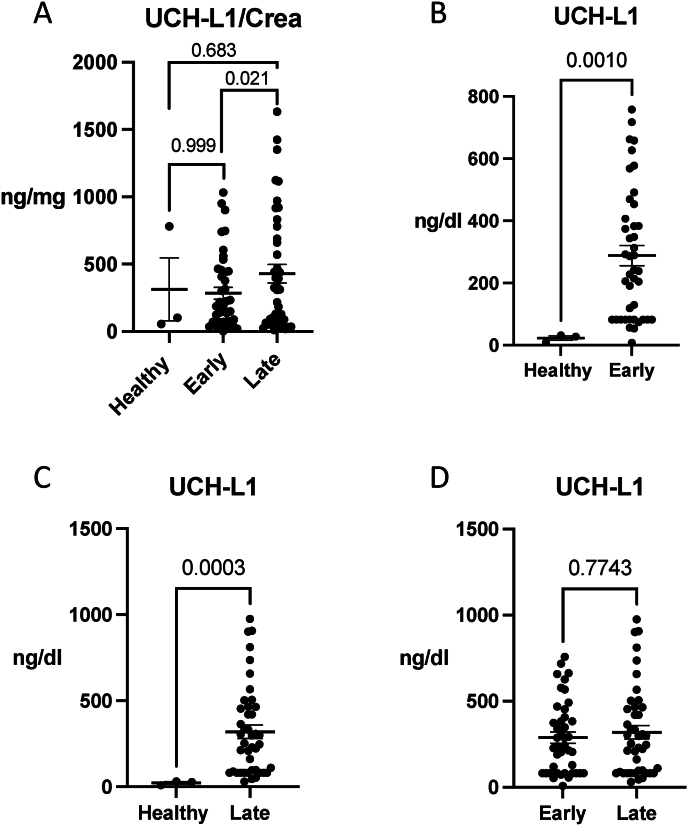
**Temporal changes of ubiquitin carboxy-terminal hydrolase L1 (UCH-L1) after acute brain injury (ABI) and comparison to healthy subjects.** A) Concentration of urine UCHL-L1 normalized to urine creatinine (UCH-L1/Crea) increased over time after ABI (p = 0.021). B) At the early timepoint ABI patients had a significantly higher concentration of UCH-L1 in urine compared to healthy subjects (p = 0.0010). C) At the late timepoint ABI patients had a significantly higher concentration of UCH-L1 in urine compared to healthy subjects (p = 0.0003). D) Over time UCH-L1 concentration did not increase in the urine of ABI patients (p = 0.7743). The data represent mean ± SEM**.** Paired Wilcoxon matched-pairs rank test when compared early and late, others Mann-Whitney *U* test.

In the healthy individuals, the mean UCH-L1/Crea concentration was 313.0 ± 406.3 ng/mg and there was not statistically significant difference between UCH-L1/Crea and early or late timepoints (Fig. 3A). UCH-L1/Crea levels in urine increased after ABI between early and late timepoints (p = 0.021) (Fig. 3A).

Interestingly, when we studied non-normalized concentrations of UCH-L1 significantly higher urine UCH-L1 concentrations were measured in ABI patients compared to healthy subjects, both early and late timepoints after ABI (p = 0.001 and p = 0.0003, respectively) (Table 2, Fig. 3B and C). Temporal change of UCH-L1 was not observed without normalization to creatinine (p = 0.77) (Fig. 3D).

**Table 2:** Concentrations of urine UCH-L1/Crea and UCH-L1 from the acute brain injury cohort (n = 46) in early and late time points and healthy controls.

Variable	Healthy (n = 3)	Early (n = 46)	Late (n = 46)	p-value
**UCH-L1/Crea (ng/mg)**				p = 0.021^a^
Mean ± SD	313.0 ± 406.3	284.0 ± 278.3	428.8 ± 441.8	
Min–Max	55.18–781.3	2.773–1033	10.63–1633	
Median (IQR)	102.5 (55.18–781.3)	179.2 (70.49–449.4)	311.5 (63.62–712.5)	
**UCH-L1 (ng/dl)**				p ≤ 0.001^b^
Mean ± SD	22.84 ± 12.27	288.3 ± 210.4	319.9 ± 258.7	
Min–Max	31.97–8.890	758.1–7.998	30.42–975.8	
Median (IQR)	27.67 (31.97–8.890)	240.2 (82.00–418.8)	253.7 (82.00–464.9)	

a Between early and late time points (p = 0.021); paired Wilcoxon matched-pairs rank test.

b Healthy controls compared to early timepoint (p = 0.0010) and late timepoint (p = 0.0003); Mann-Whitney *U* test.

### 3.5 Diagnostic properties of urine UCH-L1

**Fig. 4:**
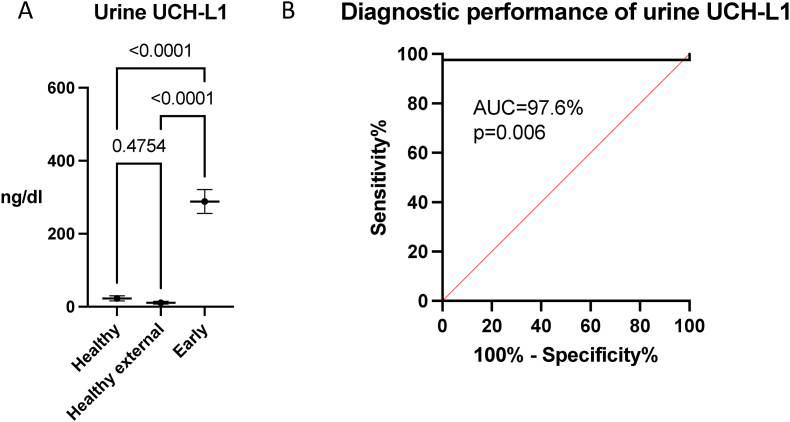
A) Urine ubiquitin carboxy-terminal hydrolase L1 (UCH-L1) concentrations in healthy subjects (n = 3) and previously published external healthy subjects (n = 10) were similar (adjusted p = 0.4754). At the early timepoint after ABI UCH-L1 is markedly increased (adjusted p < 0.0001) compared to healthy subjects. The data represent mean ± SEM. Dunnet's T3 multiple comparison test B) The receiver operating characteristic curve (ROC) testing the diagnostic performance of ubiquitin carboxy-terminal hydrolase L1 (UCH-L1) between healthy subjects (n = 3) and early acute brain injury (n = 46). Area under the curve (AUC) = 97.6% (95% confidence interval (CI) 93.0–100), p = 0.006. Sensitivity of 98% (95% CI 87.7–99.9) and specificity of 100% (95% CI 43.9–100).

The marked difference in UCH-L1 in urine between healthy subjects and ABI indicated possible diagnostic implications. To study that further, we compared and analyzed urine UCH-L1 concentrations in healthy subjects, healthy subjects from previously published data (healthy external cohort (Kohlhase et al., 2023)), and early ABI patients. In the healthy subjects in our cohort (n = 3), the urine UCH-L1 concentration was 22.84 ± 12.27 ng/dl, and in the healthy external cohort (n = 10), the urine UCH-L1 concentration was 10.96 ± 11.68 ng/dl. We further compared these data to early ABI patients’ concentration 288.3 ± 210.4 ng/dl and identified statistically significant differences between both healthy control groups (adjusted p < 0.001) (Fig. 4A). There was no statistically significant difference between our healthy cohort and the previously published cohort (p = 0.48) (Fig. 4A).

The diagnostic performance of urine UCH-L1 was evaluated at an early timepoint compared to healthy controls (n = 3). The results demonstrated excellent discriminatory ability, with an AUC of 97.6% (95% confidence interval [CI]: 93.0–100), and a p-value of 0.006. The sensitivity was 98% (95% CI: 87.7–99.9) and the specificity was 100% (95% CI: 43.9–100) (Fig. 4B). Further analysis included the imputation of previously published external healthy cohort data (n = 10, 10.96 ± 11.68 ng/dl) into the ROC analysis. This extended analysis confirmed the excellent diagnostic discriminatory ability (Supplemental Fig. S6), with improved confidence intervals for AUC and specificity due to the increased sample size. The updated results showed an AUC of 98.7% (95% CI: 96.1–100), p < 0.0001, with a sensitivity of 98% (95% CI: 87.7–99.9) and specificity of 100% (95% CI: 77.2–100). Analyses for comparing late time point to healthy controls indicated similar performance as early since late UCH-L1 concentration was higher (early 288.3 ng/dl vs. late 319.9 ng/dl, Table 2).

## 4 Discussion

In our study, we analyzed UCH-L1 concentrations in early and late urine samples from patients with IS, TBI, and aSAH to explore potential associations between biomarker levels and neurological outcomes. Our findings revealed that UCH-L1 concentrations, whether normalized to creatinine or not, did not significantly differentiate between favorable and unfavorable outcomes at the 3-month follow-up. However, significant differences were observed between healthy controls and ABI patients, highlighting the diagnostic potential of UCH-L1 for distinguishing ABI patients from healthy individuals. These results suggest that while urine UCH-L1 concentrations may not serve as a prognostic biomarker for patient outcomes, they hold promise as a non-invasive and easy access diagnostic tool to identify patients with brain injury at both early and late time points.

Elevated blood levels of UCH-L1 have been noted to be an indicator of ABI in preclinical rodent models (Liu et al., 2010) and in clinical studies (Ren et al., 2016; Papa et al., 2012; Zhang et al., 2014). The levels in serum seem to be approximately 10-fold in patients compared to healthy controls. In our study, we observed an approximately 12- to 14-fold increase in urine UCH-L1 levels compared to healthy subjects, with a trend toward increasing absolute concentrations over time, though this increase was not statistically significant. These findings align with previous studies performed from serum UCH-L1, and our results further demonstrated the accurate diagnostic performance of urine UCH-L1, suggesting its promising potential as an additional tool for diagnosing ABIs.

Levels of UCH-L1 from serum and CSF have been shown to correlate with injury severity and presence of traumatic intracranial findings (Bazarian et al., 2018; Bazarian et al., 2021; Posti et al., 2016; Diaz-Arrastia et al., 2014), and patient outcomes after TBI (Mondello et al., 2011; Anderson et al., 2020; Takala et al., 2016; Helmrich et al., 2022). Our results did not reveal a statistically significant difference in UCH-L1 concentrations across various types of brain damage; however, consistently higher concentrations were noted in the TBI group, regardless of the timing or outcome. This pattern appears influenced by outliers with low concentrations within the TBI group. These observations lend support to prior research highlighting the utility of UCH-L1 in the prognosis and diagnosis of TBI, likely attributable to the more extensive surface area of intracranial damage observed in TBI compared to other subgroups. In addition, UCH-L1 concentration has been seen to be significantly higher in those patients who did not survive during follow-up time vs. the survivors and seems to rise until death in non-survivors compared to recovered levels of UCH-L1 in survivors (Mondello et al., 2012a; Majetschak et al., 2005). From urine UCH-L1 we were not able to identify the difference between outcomes. This may be due to that neuronal-specific biomarkers tend to concentrate in urine diluting the difference that could be indicative for outcome (Kohlhase et al., 2023). Furthermore, our study noted that urine UCH-L1 levels are elevated about 10 days post-injury in ABI patients. These findings align with previous studies that have shown increased UCH-L1 concentrations persist for longer periods, with patients experiencing very unfavorable outcomes displaying significantly higher levels of UCH-L1 even more than 10 days post-injury (Lewis et al., 2010) possibly indicating ongoing neuronal injury. This suggests that urine UCH-L1 could be useful for later diagnostics, even several days after the initial injury.

Previous studies on urine UCH-L1 have been limited. However, our findings are consistent with those of Kohlhase et al. (2023), who observed that the absolute urine concentration of UCH-L1 was higher in patients with brain injuries compared to healthy controls (Kohlhase et al., 2023). They found that urine UCH-L1 concentration in healthy subjects was 10.96 ± 11.68 ng/dl (n = 10), while in our study the concentration was slightly higher 22.84 ± 12.27 ng/dl. Notably, we detected higher urine UCH-L1 concentration in ABI patients as well. The higher UCH-L1 values observed in our study, compared to those reported by Kohlhase et al. (2023), may be due to the use of different detection kits, which could lead to variability in the measured concentrations (Commissioner O of the. et al., 2020). Additionally, Kohlhase et al. (2023) excluded 16 patients due to saturation issues, potentially omitting individuals with higher UCH-L1 levels from their analysis (Commissioner O of the. et al., 2020). There was also significant difference in the studied cohorts. Kohlhase et al. (2023) studied IS and intracerebral hemorrhage patients (Commissioner O of the. et al., 2020), while our study included patients with IS, aSAH, and acute subdural hematomas. Furthermore, our cohort was 13 years younger on average. These differences likely account for the discrepancies in UCH-L1 measurements between the studies. It is interesting that upon examining the creatinine-normalized UCH-L1 concentration, their findings indicated that the diagnostic significance of UCH-L1 diminishes. A similar result was found in our study. This result elevates the interest in UCH-L1 without normalization to creatine. A continuous challenge in urine biomarkers has been the influence of external factors on urine, such as the timing of sample collection, medications, kidney function, and dietary factors (Seol et al., 2020). Despite these challenges, considering this study and previous evidence, urine UCH-L1 can be considered a significant neuronal marker worthy of further research.

### 4.1 Limitations

Our study has certain limitations that need to be considered while interpreting our findings. Firstly, the uneven distribution of patients with favorable and unfavorable outcomes in our sample may lead to bias in the comparative analysis. We had a modest sample size of only 46 participants which may limit the generalizability and statistical power of our findings. However, we have addressed this issue by analyzing urinary UCH-L1 concentrations at two different timepoints post-admission. Additionally, the small sample size of the study did not allow for multivariate analysis, which could account for potential confounding factors. However, statistical analyses showed no significant differences in age or sex between groups. Nonetheless, to ensure the validity of our results, it is necessary to replicate this study with a larger cohort. Furthermore, the number of our control patients was small, but the results were consistent with previous studies.

Thirdly, the inclusion of three distinct types of acute brain injuries (aSAH, IS, and TBI) adds a layer of complexity due to the inherent heterogeneity in disease pathologies and patient responses. Our cohort exhibits significant variation in the location of brain injury, which, in turn, complicates the comparison of patients’ functional outcomes in relation to the brain injury.

While our results suggest that UCH-L1 concentrations may serve as a diagnostic biomarker irrespective of injury type, a more exhaustive exploration across individual injury types and severity may yield more profound insights into the specific roles and implications of UCH-L1.

## 5 Conclusion

Our study indicates that urine UCH-L1 is a promising diagnostic biomarker for patients with ABI, showing significantly higher levels compared to healthy controls, irrespective of the type of brain injury. However, UCH-L1 concentrations in urine did not differentiate between favorable and unfavorable outcomes, highlighting its diagnostic utility but limited prognostic value. These findings encourage larger studies and increase interest in developing easily accessible biofluid tests for diagnosing ABIs.

## Author contributions:

The study was conceptualized, designed, and grant funded by J.K. Laboratory work was carried out by S.H., J.K., A.S., F.K., and S.K. Statistical analyses were conducted by Y.C. (biostatistician), J.K., and S.H. Assessing patient outcomes was the responsibility of M.R., J.K., S.R., and F.K. The results were interpreted, and the initial manuscript and figures were drafted by S.H., A.S., and J.K. The manuscript was critically reviewed, edited, and revised by A.J., A. Sr., C.B., J.F., T.R., S.B.L., Jo.F., R.T., J.P.P., S.R., F.K., J.P., Y.C., M.J., M.R., J.R., and E.C. All authors have read and approved the final version of the manuscript for submission.

## Data availability

The anonymized data from this study can be made available upon request to qualified researchers who have obtained appropriate institutional review board (IRB) approval. Requests should be directed to the corresponding author (JK).

## Funding

Funding was received for this work by JK from the Sigrid Juselius Foundation and the Finnish Medical Foundation. AS received funding from the Sigrid Juselius Foundation and the Maire Taponen Foundation. SH received funding from the Sigrid Juselius Foundation. JPP received funding from the Research Council of Finland (grant 60063).

## Declaration of competing interest

The authors declare the following financial interests/personal relationships which may be considered as potential competing interests: Janne Koskimaki reports financial support was provided by Sigrid Jusélius Foundation. Janne Koskimaki reports financial support was provided by 10.13039/100008723Finnish Medical Foundation. Santtu Hellstrom reports financial support was provided by Sigrid Jusélius Foundation. Antti Sajanti reports financial support was provided by Sigrid Jusélius Foundation. Antti Sajanti reports financial support was provided by Maire Taponen Foundation. Jussi Posti reports financial support was provided by Research Council of Finland. If there are other authors, they declare that they have no known competing financial interests or personal relationships that could have appeared to influence the work reported in this paper.
