# Supplementary material for: Diagnostic and prognostic performance of urine ubiquitin carboxy-terminal hydrolase L1 across multiple acute brain injury types – A longitudinal prospective cohort study
Source: Brain Spine. 2024 Dec 24;5:104173. doi: 10.1016/j.bas.2024.104173 (PMC11743582; doi:10.1016/j.bas.2024.104173)
Supplement: Multimedia component 1 [file mmc1.docx]

**Supplementary material**

**Diagnostic and prognostic performance of urine ubiquitin carboxy-terminal hydrolase L1 across multiple acute brain injury types – A longitudinal prospective cohort study**

Santtu Hellström MD^1^, Antti Sajanti MD^1^, Aditya Jhaveri BS^2^, Abhinav Srinath PhD^2^, Carolyn Bennett MPhil ^2^, Ying Cao MS^3^, Fredrika Koskimäki MD^5^, Johannes Falter MD^4^, Janek Frantzén MD, PhD^1^, Seán B. Lyne MD^6^, Tomi Rantamäki PhD^7, 8^, Riikka Takala MD, PhD^9^, Jussi P. Posti MD, PhD^1^, Susanna Roine MD, PhD^5^, Sulo Kolehmainen BS^10^, Miro Jänkälä MD^11^, Jukka Puolitaival MD^11^, Romuald Girard PhD^2^, Melissa Rahi MD, PhD^1^, Jaakko Rinne MD, PhD^1^, Eero Castrén MD, PhD^10^, Janne Koskimäki MD, PhD^1, 10, 11^

**Author affiliations:**

^1^Neurocenter, Department of Neurosurgery, Turku University Hospital and University of Turku, P.O. Box 52, FI-20521, Turku, Finland.

^2^Neurovascular Surgery Program, Section of Neurosurgery, The University of Chicago Medicine and Biological Sciences, Chicago, IL 60637, US.

^3^Department of Radiation Oncology, Kansas University Medical Center, Kansas City, KS 66160, USA

^4^Department of Neurosurgery, University Medical Center of Regensburg, Regensburg, 93042, Germany

^5^Neurocenter, Acute Stroke Unit, Turku University Hospital, P.O. Box 52, FI-20521, Turku, Finland

^6^Department of Neurosurgery, Brigham and Women’s Hospital, Harvard Medical School, Boston, MA, US.

^7^Laboratory of Neurotherapeutics, Drug Research Program, Division of Pharmacology and Pharmacotherapy, Faculty of Pharmacy, University of Helsinki, P.O. Box 56, FI-00014 Helsinki, Finland

^8^SleepWell Research Program, Faculty of Medicine, University of Helsinki, P.O. Box 63, FI-00014 Helsinki, Finland

^9^Perioperative Services, Intensive Care and Pain Medicine and Department of Anaesthesiology and Intensive Care, Turku University Hospital and University of Turku, P.O. Box52, FI-20521, Turku, Finland

^10^Neuroscience Center, HiLIFE, University of Helsinki, P.O. Box 63, FI-00014 Helsinki, Finland.

^11^Department of Neurosurgery, Oulu University Hospital, Box 25, 90029 OYS, Finland.

**Correspondence to**: Janne Koskimäki, MD, PhD, Associate Professor

**Full address**: Neurocenter, Department of Neurosurgery, Turku University Hospital and University of Turku, P.O. Box 52 (Hämeentie 11), FI-20521, Turku, Finland.

**E-mail:** jankosk@utu.fi

**Keywords:** brain injury; UCHL1; urine; biomarker; outcome; prognosis; stroke; traumatic brain injury

**Supplementary results:**

**Demographics and clinical characteristics of enrolled patients**

There were 22 patients in our cohort that suffered from aSAH. Age of patients varied from 34 to 75 years with mean age being 55.8 ± 12.5 years in the favorable group and 58.9 ± 14.6 in the unfavorable group. In the outcome subgroups, there were six males (46.2%) and 7 (53.8%) females in the favorable group and three males (33.3%) and six (66.7%) males in the unfavorable group **(Suppl. Table 1.)**. In the IS subcohort, there were 16 patients with an age range of 37 to 71 years. The mean age was 60.4 ± 11.4 years in the favorable group and 55.0 ± 21.2 in the unfavorable group. There were eight males (57.1%) and six females (42.9%) in the favorable group and in the unfavorable group both patients (n=2) were males. In our TBI subcohort, we had eight patients. Age varied from 23 to 71 years, with mean age being 68.0 ± 23.3 years in the favorable group and 60.0 ± 12.3 years in the unfavorable group. There were seven (87.5%) males and one (12.5%) female in the group (**Suppl. Table 1**).

We also focused on the demographical and clinical characteristics of the ABI patients to explore the correlations between them and the concentrations of UCH-L1. Using the Pearson correlation, we found that neither age nor sex significantly affects the UCH-L1 levels in the urine of ABI patients (p=0.8169 and p=0.6522) (**Suppl. Figure 1 A and B**).

**Supplemental Figure 1. Correlation analysis of acute brain injury (ABI) patients' age and sex with UCH-L1.** A) No significant correlation was identified between age and UCH-L1 (Pearson correlation: r = 0.03550, 95% CI: -0.2608 to 0.3256, R² = 0.001260, p = 0.8169). B) No significant correlation was identified between sex and UCH-L1 (Pearson correlation: r = -0.06904, 95% CI: -0.3554 to 0.2291, R² = 0.004767, p = 0.6522).

Furthermore, we made a correlation analysis between some well-known clinical features of different ABIs. We aimed to identify potential relationships that might aid in understanding the biomarker’s behavior in response to different injury types and outcomes.

**Ischemic Stroke**

For the IS group, we examined the connection between UCH-L1 level and different features of stroke that included stroke volume (early and late sample), stroke type, and stroke location in the arterial system (**Suppl. Figure 2**). We found no significant association between stroke volume and UCH-L1 levels neither in the early or late samples (p=0.8852 and p=0.7894, respectively) (**Suppl. Figure 2A and B**). Interestingly, we found a clear trend between UCH-L1 concentration and different types of IS (p=0.0666). Cryptogenic stroke was associated with the highest levels and cardiogenic stroke with the lowest. Thrombosis-based stroke had levels in between cryptogenic and cardiogenic stroke. There was no statistically significant correlation between the different locations of the stroke and UCH-L1 levels (p=0.4979) (**Suppl. Figure 2D**).

**Supplemental Figure 2.** **Correlations of the clinical features of ischemic stroke and UCH-L1. Type of the ischemic stroke (IS) showed a clear trend between type and UCH-L1 concentration.** A) Pearson correlation (r = 0.03928, 95% CI: -0.4655 to 0.5248, R² = 0.001543, p = 0.8852). B) Pearson correlation (r = 0.07539, 95% CI: -0.4544 to 0.5658, R² = 0.005683, p = 0.7894). C) Pearson correlation (r = 0.4694, 95% CI: -0.03423 to 0.7830, R² = 0.2204, p = 0.0666). D) Pearson correlation (r = -0.1829, 95% CI: -0.6222 to 0.3440, R² = 0.03344, p = 0.4979).

**Aneurysmal subarachnoid hemorrhage**

In our study, the clinical features investigated for aSAH included clinical grade, neurological status, severe complications, and anatomical location. The modified Fisher scale is a widely accepted radiological grading method for aSAH (1). Our analysis explored the relationship between the modified Fisher scale scores and UCH-L1 levels in urine. We found no significant correlation between these variables (p=0.5721) (**Suppl. Figure 3A**). Secondly, we examined the association between neuroworsening and UCH-L1 levels in urine. Neuroworsening was defined by the presence of one or more of the following criteria: a spontaneous reduction in the Glasgow Coma Scale (GCS) motor score by two points or more from the previous examination; the emergence of new pupillary reactivity loss or the development of pupillary asymmetry of 2mm or greater; and a deterioration in neurological or CT findings that necessitated immediate medical or surgical intervention. Our analysis revealed no significant correlation between neuroworsening and urine UCH-L1 concentrations (**Suppl. Figure 3B**). Additionally, we examined the correlation between UCH-L1 levels and the occurrence of delayed cerebral ischemia (DCI). Interestingly, this analysis found a statistically significant and moderately negative correlation (p=0.0202), suggesting an inverse relationship between UCH-L1 levels and DCI (**Suppl. Figure 3C**). Different anatomical locations of the hemorrhage showed no significant correlation with UCH-L1 concentration (p=0.598) (**Suppl. Figure 3D)**.

**Supplemental Figure 3. Correlations of the clinical features of aneurysmal subarachnoid hemorrhage (aSAH) and UCH-L1. Clinical delayed cerebral ischemia (DCI) correlated with UCH-L1 levels.** A) Pearson correlation (r = 0.1190, 95% CI: -0.3186 to 0.5148, R² = 0.01417, p = 0.5978). B) Pearson correlation (r = 0.1090, 95% CI: -0.3386 to 0.5164, R² = 0.01189, p = 0.6380) C) Pearson correlation (r = -0.5026, 95% CI: -0.7677 to -0.09056, R² = 0.2526, p = 0.0202). D) Pearson correlation (r = 0.1307, 95% CI: -0.3189 to 0.5324, R² = 0.01710, p = 0.5721).

**Traumatic brain injury**

For the TBI, we investigated the correlation between UCH-L1 concentration and the volume of acute subdural hematoma (aSDH), and also the midline shift in millimeters and the neuroworsening. Interestingly, we found that UCH-L1 levels were significantly higher when the volume of the aSDH was higher. There was a strong positive relationship between these two factors (p=0.0011) (**Suppl. Figure 4A**). The midline shift did not correlate with the UCH-L1 levels (p=0.91) (**Suppl. Figure 4B**). Similar to the aSAH, neuroworsening didn’t cause a significant change in the UCH-L1 levels in the TBI patients (p=0.57) (**Suppl. Figure 4C**).

**Supplemental Figure 4.** **Correlations of the clinical features of traumatic brain injury (TBI) and UCH-L1. TBI patients’ acute subdural hematoma volume correlated with UCH-L1 levels** A) Pearson correlation (r = 0.9219, 95% CI: 0.6201 to 0.9860, R² = 0.8499, p = 0.0011). B) Pearson correlation (r = -0.04776, 95% CI: -0.7279 to 0.6798, R² = 0.002281, p = 0.9106). C) Pearson correlation (r = 0.2391, 95% CI: -0.5599 to 0.8077, R² = 0.05717, p = 0.5685).

**Temporal and outcome-dependent patterns of UCH-L1 in urine of ABI patients**

In our supplementary analyses of concentration, using the Kruskal-Wallis test, we explored the effect of the UCH-L1 on patient outcomes across specific disease groups, considering both early and late sample time points, as well as the designated outcomes. We went through four different situations: early favorable, late favorable, early unfavorable, and late unfavorable (**Suppl. Figure 5 A and D**). In the early samples of the favorable outcome group, the UCH-L1 concentration did not show a statistical difference between the groups (p=0.34) (**Suppl. Figure 5A**). Similarly, the late measurements didn’t show significant changes in UCH-L1 levels (p=0.35) (**Suppl. Figure 5B**). Neither in more severely affected patients there were no statistically significant differences between early unfavorable and late unfavorable groups (p=0.14 and p=0.66, respectively) (**Suppl. Figure 5C and D**).

**Supplemental Figure 5. UCH-L1 profiles in urine across different acute brain injuries.** Considering different time points and outcomes, we did not observe statistically significant differences in the concentration of UCH-L1 in urine. A) Early Favorable Group (p=0.34), B) Late Favorable Group (p=0.35), C) Early Unfavorable Group (p=0.14), and D) Late Unfavorable Group (p=0.66). Kruskal-Wallis test. Data represent mean ± SEM (ng/dl).

**Enhanced Diagnostic Utility of UCH-L1 with External Healthy Cohort Data Integration**

We explored an extended analysis of the diagnostic properties of UCH-L1 levels, including data from a previously published external healthy cohort (n=10, mean UCH-L1 concentration 10.96 ± 11.68 ng/dl)(2). We aimed to assess how the inclusion of additional control data impacts the biomarker’s diagnostic performance. We generated the receiver operating characteristic (ROC) curve analysis to measure the ability of UCH-L1 concentration to differentiate patients with ABI and healthy cohorts (**Suppl. Figure 6)**. The area under the curve (AUC) for our analysis reached 98.7% (95% confidence interval [CI]: 96.1-100) demonstrating an excellent level of diagnostic discrimination. The sensitivity of the UCH-L1 biomarker was high at 98% (95% CI: 87.7-99.9), with specificity also calculated at 100% (95% CI: 77.2-100). The p-value was <0.0001 indicating a highly significant statistical difference affirming UCH-L1’s role as a diagnostic marker.

**Supplemental Figure 6.** **Diagnostic properties of urine UCH-L1 with previously published external healthy cohort data.** The diagnostic performance of urine UCH-L1 was also evaluated with the inclusion of previously published external healthy cohort data (n=10, 10.96 ± 11.68 ng/dl) into the ROC analysis. This extended analysis confirmed the excellent diagnostic discriminatory ability, with improved confidence intervals for AUC and specificity due to the increased sample size. The results showed an area under the curve (AUC) of 98.7% (95% confidence interval [CI]: 96.1-100), p<0.0001, with sensitivity of 98% (95% CI: 87.7-99.9) and specificity of 100% (95% CI: 77.2-100).

**Supplementary discussion**

By examining the effect of the demographical and clinical characteristics on the levels of urine UCH-L1, we found no significant association between the levels and age or sex of the ABI patients. This finding supports the potential utility of UCH-L1 a biomarker for brain injury, independent of these demographic factors reinforcing the biomarker’s reliability and consistency across a diverse patient population.

ROC curve analysis highlights the diagnostic performance of urine UCH-L1 between ABI patients and healthy patients affirming the utility of urine samples in the biomarker studies in the field of ABI’s. The disease characteristics of various brain injuries provided additional insights into diverse clinical features. Particularly notable was the correlation between the volume of aSDH and UCH-L1 levels in cases of TBI. This finding further supports existing knowledge regarding the role of UCH-L1 as a reliable diagnostic biomarker for TBI(3,4). While most examined features did not exhibit significant correlations with UCH-L1 levels, the noteworthy association with DCI slightly controversially showed that lower levels of UCH-L1 might predict a higher risk of DCI. However, further investigation is essential to confirm these results.

In summary, our supplementary analysis concludes that the use of UCH-L1 as a diagnostic biomarker is not limited to a specific patient group or demographic cohort; rather, the biomarker can be broadly utilized as a diagnostic tool for brain injuries. Future studies involving a broader patient cohort are necessary to validate these findings and to identify potential factors concerning distinct disease groups.

**References**

1. Zhao D, Li Y, Cui J. The factors associated with the modified Fisher grade in patients with aneurysmal subarachnoid hemorrhage. Front Physiol. 2024 Jul 17;15:1373925.

2. Kohlhase K, Frank F, Wilmes C, Koerbel K, Schaller-Paule MA, Miles M, et al. Brain-specific biomarkers in urine as a non-invasive approach to monitor neuronal and glial damage. Eur J Neurol. 2023;30(3):729–40.

3. Papa L, Lewis LM, Silvestri S, Falk JL, Giordano P, Brophy GM, et al. Serum levels of Ubiquitin C-terminal Hydrolase (UCH-L1) distinguish mild traumatic brain injury (TBI) from trauma controls and are elevated in mild and moderate TBI patients with intracranial lesions and neurosurgical intervention. J Trauma Acute Care Surg. 2012 May;72(5):1335–44.

4. Shahjouei S, Sadeghi-Naini M, Yang Z, Kobeissy F, Rathore D, Shokraneh F, et al. The diagnostic values of UCH-L1 in traumatic brain injury: A meta-analysis. Brain Inj. 2018 Jan 2;32(1):1–17.
