# Supplementary material for: Diagnostic and prognostic performance of urine ubiquitin carboxy-terminal hydrolase L1 across multiple acute brain injury types – A longitudinal prospective cohort study
Source: Brain Spine. 2024 Dec 24;5:104173. doi: 10.1016/j.bas.2024.104173 (PMC11743582; doi:10.1016/j.bas.2024.104173)
Supplement: Multimedia component 2 [file mmc2.docx]

| **Supplemental table I. Factors associated with outcome in univariate analysis.** | | | | |
| --- | --- | --- | --- | --- |
|  |  |  |  |  |
| **Aneurysmal subarachnoid hemorrhage** | |  |  |  |
|  | **Favorable (mRS 0-3, n=13)** | **Unfavorable (mRS 4-6, n=9)** | **p-value** |  |
|  |  |  |  |  |
| **Age in years** |  |  | 0.5968 |  |
| Mean±SD | 55.8±12.5 | 58.9±14.6 |  |  |
| Min–Max | 38.0–75.0 | 34.0–74.0 |  |  |
| Median (IQR) | 59.0 (43.0–65.0) | 65.0 (47.0–70.0) |  |  |
| **Sex** |  |  | 0.5476 |  |
| Male | 6 (46.2) | 3 (33.3) |  |  |
| Female | 7 (53.8) | 6 (66.7) |  |  |
| **mRS** |  |  | <.0001 |  |
| 1 | 13 (100) |  |  |  |
| **GCS scene** |  |  | 0.1200 |  |
| Mean±SD | 12.8±3.7 | 9.7±5.5 |  |  |
| Min–Max | 5.0–15.0 | 3.0–15.0 |  |  |
| Median (IQR) | 15.0 (13.0–15.0) | 12.0 (4.0–15.0) |  |  |
| **Neuroworsening** |  |  | 0.0180 |  |
| No | 8 (61.5) | 1 (11.1) |  |  |
| Yes | 5 (38.5) | 8 (88.9) |  |  |
| **Modified Fisher Scale** |  |  | 0.3325 |  |
| Mean±SD | 3.5±0.8 | 3.8±0.7 |  |  |
| Min–Max | 2.0–4.0 | 2.0–4.0 |  |  |
| Median (IQR) | 4.0 (3.0–4.0) | 4.0 (4.0–4.0) |  |  |
| **H&H** |  |  | 0.0205 |  |
| Mean±SD | 2.3±1.4 | 3.8±1.3 |  |  |
| Min–Max | 1.0–5.0 | 2.0–5.0 |  |  |
| Median (IQR) | 2.0 (1.0–3.0) | 4.0 (3.0–5.0) |  |  |
| **Location of Aneurysm** |  |  | 0.2507 |  |
| ACA | 1 (7.7) |  |  |  |
| AcomA | 5 (38.5) | 3 (33.3) |  |  |
| MCA | 1 (7.7) | 3 (33.3) |  |  |
| BAS | 1 (7.7) |  |  |  |
| PcomA |  | 2 (22.2) |  |  |
| PICA | 1 (7.7) |  |  |  |
| ICA | 4 (30.8) | 1 (11.1) |  |  |
| **Diagnosis of Acute Hydrocephalus** |  |  | 0.5121 |  |
| No | 9 (69.2) | 5 (55.6) |  |  |
| Yes | 4 (30.8) | 4 (44.4) |  |  |
| **Clinical DCI** |  |  | 0.1324 |  |
| No | 11 (84.6) | 5 (55.6) |  |  |
| Yes | 2 (15.4) | 4 (44.4) |  |  |
|  |  |  |  |  |
| **Ischemic stroke** | | |  |  |
|  | **Favorable (mRS 0-3, n=14)** | **Unfavorable (mRS 4-6, n=2)** | **p-value** |  |
|  |  |  |  |  |
| **Age in years** |  |  | 0.5703 |  |
| Mean±SD | 60.4±11.4 | 55.0±21.2 |  |  |
| Min–Max | 37.0–74.0 | 40.0–70.0 |  |  |
| Median (IQR) | 64.0 (51.0–71.0) | 55.0 (40.0–70.0) |  |  |
| **Sex** |  |  | 0.2416 |  |
| Male | 8 (57.1) | 2 (100) |  |  |
| Female | 6 (42.9) |  |  |  |
| **Neuroworsening** |  |  | 0.4677 |  |
| No | 11 (78.6) | 2 (100) |  |  |
| Yes | 3 (21.4) |  |  |  |
| **Type of infarction** |  |  | 0.2301 |  |
| Cardiogenic | 4 (28.6) |  |  |  |
| Thrombosis | 5 (35.7) |  |  |  |
| Cryptic | 5 (35.7) | 2 (100) |  |  |
| **Volume of infarction (ml)** |  |  | 0.5488 |  |
| Mean±SD | 10.3±16.0 | 58.5±79.6 |  |  |
| Min–Max | 0.0–46.8 | 2.2–114.8 |  |  |
| Median (IQR) | 3.7 (0.4–6.4) | 58.5 (2.2–114.8) |  |  |

| **Traumatic brain injury** | | |  |  |
| --- | --- | --- | --- | --- |
|  | **Favorable (mRS 0-3, n=3)** | **Unfavorable (mRS 4-6, n=5)** | **p-value** |  |
|  |  |  |  |  |
| **Age in years** |  |  | 0.4295 |  |
| Mean±SD | 68.0±24.3 | 60.0±12.3 |  |  |
| Min–Max | 23.0–71.0 | 42.0–71.0 |  |  |
| Median (IQR) | 54.0 (23.0–71.0) | 65.0 (47.5–70.0) |  |  |
| **Sex** |  |  | 0.4816 |  |
| Male | 3 (100) | 4 (80) |  |  |
| Female | 0 (0) | 1 (20) |  |  |
| **Neuroworsening** |  |  | 0.7246 |  |
| No | 1 (33.3) | 1 (20) |  |  |
| Yes | 2 (66.6) | 4 (80) |  |  |
| **Thickness of aSDH (mm)** |  |  | 0.2805 |  |
| Mean±SD | 12.67±7.5 | 8.1±3.68 |  |  |
| Min-Max | 5.0-20.0 | 4.5–12.0 |  |  |
| Meadian (IQR) | 13.0 (5.0–20.0) | 7.0 (4.75–12.0) |  |  |
| **Midline shift (mm)** |  |  | 0.5008 |  |
| Mean±SD | 8.33±7.64 | 5.6±3.4 |  |  |
| Min–Max | 0.0–15.0 | 0.0–9.0 |  |  |
| Median (IQR) | 10.0 (0.0–15.0) | 7.0 (2.5–8.0) |  |  |

mRS=Modified Ranking Scale, H&H=Hunt and Hess Classification

ACA=Anterior Cerebral Artery, AcomA=Anterior Communicans Artery, MCA=Middle Cerebral Artery, BAS=Basilar Artery, PICA=Posterior Inferior Cerebellar Artery, ICA=Internal Carotic Artery
DCI=Delayed Clinical Ischemia, GCS=Glascow Coma Scale
